# The Effect of Gliding Arc Discharge Low-Temperature Plasma Pretreatment on Blueberry Drying

**DOI:** 10.3390/foods14081344

**Published:** 2025-04-14

**Authors:** Pengpeng Yu, Wenhui Zhu, Yu Qiao, Xiaonan Yang, Lixin Ma, Yankai Cai, Jianrong Cai

**Affiliations:** 1 School of Agricultural Engineering, Jiangsu University, Zhenjiang 212013, China; 2 School of Food and Biological Engineering, Jiangsu University, Zhenjiang 212013, China; 15393060631@163.com (W.Z.); qiaoyuqiaoyu@126.com (Y.Q.); yxnfight@163.com (X.Y.); lixinma99@163.com (L.M.); 2222018001@stmail.ujs.edu.cn (Y.C.)

**Keywords:** low-temperature plasma, blueberry, drying kinetics, quality changes

## Abstract

This study evaluates the effects of gliding arc discharge low-temperature plasma (GAD-LTP) pretreatment on the drying performance and quality attributes of blueberries. Fresh blueberries were pretreated under varying conditions—treatment durations of 6 s, 12 s, and 18 s and power levels of 300 W, 600 W, and 900 W—prior to convective hot air drying at 65 °C. Results demonstrate that plasma pretreatment significantly reduced drying time, with an 18 s treatment at 900 W reducing drying time by 31.25%. Moisture diffusion coefficients increased with both treatment duration and power. Under optimal conditions, total phenolic content improved by up to 33.47%, while anthocyanin retention initially declined then recovered, reaching a 7.9% increase over the control. However, plasma-treated samples exhibited darker color due to surface etching and oxidation. Rehydration capacity improved, with a maximum enhancement of 27.94%. Texture analysis indicated increased hardness and decreased adhesiveness and chewiness in treated samples. Overall, GAD-LTP pretreatment enhances drying efficiency and preserves bioactive compounds in dried blueberries, offering a scalable approach for industrial application.

## 1. Introduction

Blueberries (*Vaccinium* spp.) are known for their delicate texture, balanced sweet-tart flavor, and distinctive taste, making them one of the few naturally blue foods. They are highly nutritious, particularly rich in phenolic compounds such as anthocyanins, flavan-3-ols, proanthocyanidins, and flavonols, which exhibit anti-aging properties and may reduce the risk of cardiovascular diseases and cancer. Research indicates that blueberries contain the highest levels of anthocyanins among all fruits and vegetables, earning them the title of the “king of berries” [1,2,3].

However, their high moisture content makes fresh blueberries highly perishable. Post-harvest physiological and biochemical activities affect their storage stability and disease resistance. Their soft texture increases susceptibility to mechanical damage during transportation, while prolonged storage results in significant water loss, reducing consumer acceptance. Due to these factors, fresh blueberries are difficult to store at room temperature, necessitating various processing methods such as blueberry wine production, juice extraction, and drying to extend shelf life [4].

Drying is one of the most common processing techniques, as dried blueberries are compact, easy to transport and store, and retain a unique flavor favored by consumers [5]. Among various drying methods, hot air drying is the most widely used due to its low cost and industrial feasibility [6]. However, the waxy cuticle on the blueberry surface slows moisture diffusion, leading to prolonged drying times and significant nutrient loss. To address this, pretreatment methods are commonly used to enhance moisture diffusion, including chemical drying agents, perforation pretreatment, ultrasonic pretreatment [7,8], and freeze–thaw pretreatment [9].

Chemical pretreatment typically involves alkaline solutions such as ethyl oleate, sodium hydroxide (NaOH), or sodium carbonate (Na_2_CO_3_) to facilitate moisture removal [10]. However, these chemicals pose food safety risks and other concerns, leading to their gradual prohibition in food processing [11]. Perforation pretreatment, which creates micro-perforations in the waxy cuticle using lasers or mechanical methods, enhances moisture diffusion but can cause surface cracking during drying, resulting in nutrient loss and microbial contamination. Additionally, its high cost and complexity limit large-scale application [12]. Ultrasonic pretreatment accelerates moisture evaporation and heat transfer by disrupting cell walls and membrane structures through cavitation effects. However, it can negatively impact blueberry color, reduce antioxidant properties, and increase processing costs, restricting widespread adoption [13].

Although traditional pretreatments can shorten drying time, they have notable limitations [8]. Recently, low-temperature plasma has gained attention due to its residue-free nature, low temperature, and effective etching capabilities. Low-temperature plasma is a partially ionized gas generated at ambient temperature by applying high voltage to excite gas molecules. Its main components include electrons, ions, free radicals, excited atoms and molecules, as well as reactive oxygen species (e.g., O, O_3_, OH) and nitrogen species (e.g., NO, NO_2_) [14]. The type and concentration of these active components depend on factors such as discharge power, treatment duration, working gas, and reactor design. In food processing, low-temperature plasma interacts with polysaccharides, proteins, and lipids on food surfaces through these reactive species, causing surface etching, structural modifications, and changes in enzyme activity, thereby influencing drying efficiency, nutrient retention, color, and texture. For blueberries, especially due to the presence of the waxy cuticle, low-temperature plasma has a significant effect. The waxy cuticle on blueberries, composed mainly of long-chain fatty acids and wax, slows down moisture diffusion. Reactive oxygen species generated by gliding arc discharge, such as oxygen free radicals (O, O_3_), react with the waxy surface, causing oxidative damage and creating micro-pores or cracks. These structural changes effectively increase the pathways for moisture diffusion, promoting the movement of moisture from inside the blueberry to the surface. Moreover, hydroxyl radicals (OH) attack the polysaccharides or proteins in the blueberry cell wall, altering their chemical structure and enhancing the permeability of the cell wall, further accelerating moisture diffusion. Nitrogen oxides (NO, NO_2_) affect the cell membrane by changing its physical and chemical properties, increasing its fluidity and permeability, and thus improving the transport of moisture within the blueberry cells.

In comparison to traditional discharge methods, such as corona discharge and glow discharge, which are less suitable for large-scale fruit and vegetable pretreatment due to instability and the need for low-pressure or vacuum conditions, a wide variety of plasma torches, including dielectric barrier discharge (DBD), radio frequency (RF) discharge, gliding arc discharge, plasma jet, and atmospheric pressure plasma (APP), offer greater industrial applicability [15]. These plasma sources, particularly gliding arc discharge low-temperature plasma, can generate stable plasma at ambient pressure and effectively etch the surface of blueberries, enhancing moisture diffusion and reducing nutrient loss caused by prolonged exposure to high temperatures.

Gliding arc discharge, in contrast, offers ease of operation and industrial scalability, attracting research interest in fruit and vegetable drying. Ashtiani et al. found that gliding arc plasma pretreatment reduced grape drying time by approximately 11 h compared to controls while preserving vitamin C and total phenolic content [16]. Zhang et al. reported that this method enhanced drying rates, antioxidant capacity, and pigment extraction yield in chili peppers [17]. Similarly, Tabibian et al. demonstrated that it significantly reduced saffron drying time while improving pigment retention and antioxidant activity [18].

These findings suggest that gliding arc discharge low-temperature plasma is a promising pretreatment for drying waxy-coated fruits and vegetables. Conducted under ambient temperature and pressure, it is particularly suitable for delicate fruits like blueberries, which are prone to mechanical damage. This study applies low-temperature plasma technology to blueberry drying to reduce drying time, improve production efficiency, and enhance nutrient retention while ensuring industrial scalability. To date, no studies have explored its application in blueberry drying. If successfully developed and scaled, this technology could produce high-value dried blueberries with strong market potential, particularly in premium segments.

## 2. Materials and Methods

### 2.1. Materials and Equipment

Freshly harvested Yi Ke Mei-1 blueberries were obtained from a plantation in Xishuangbanna, Yunnan. In the laboratory, berries of uniform size and color, free from cracks, surface defects, or pest damage, were manually selected. The final selection had an average weight of 2.00 ± 0.05 g and an average diameter of 17.00 ± 0.30 mm. The blueberries were washed in cold water to remove impurities and gently blotted dry with absorbent paper.

**Figure 1:**
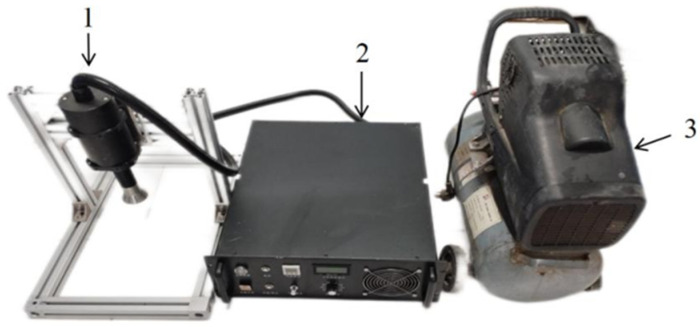
Schematic diagram of the gliding arc discharge low-temperature plasma system; 1—plasma nozzle; 2—plasma power supply; 3—booster pump.

The gliding arc low-temperature plasma device used in this study is shown in Figure 1. It comprises three main components: a plasma nozzle, a plasma power supply, and a booster pump. The booster pump generates airflow, enabling plasma ejection from the nozzle, while the power supply allows for adjustable parameters, with power being the primary variable. The low-temperature plasma pretreatment system used in this study is the XBS-75 model, produced by Shenzhen Xibosi Automation Equipment Co., Ltd. (Shenzhen, China) This system features an adjustable power range from 300 W to 900 W, allowing control over treatment intensity by varying the power. It operates using a high-frequency power supply with a working frequency of 20 kHz, which generates a stable low-temperature plasma. The system uses air as the gas source, with a flow rate ranging from 2 to 5 L/min.

To ensure accurate and consistent results, environmental variables such as ambient temperature, humidity, and gas composition were carefully monitored and controlled during the experiments. The ambient temperature was maintained between 20 °C and 25 °C to ensure stable plasma generation, while humidity levels were kept within a range of 40% to 60%, as fluctuations in humidity can affect the effectiveness of plasma treatment. Additionally, the composition of the gas was ensured to be consistent, with air being the primary gas used, as variations in gas composition can influence the generation of reactive oxygen species (such as O_3_ and OH).

During the treatment process, the temperature was typically maintained between 30 °C and 90 °C to prevent the effects of high temperatures on the samples. The pretreatment time was adjustable, ranging from 6 to 30 s, to accommodate different experimental requirements. This GAD-LTP system generates reactive oxygen species through gliding arc discharge, which enhances the moisture diffusivity of the blueberries, thereby accelerating the drying process while preserving their nutritional content.

### 2.2. Experimental Design of Gliding Arc Low-Temperature Plasma Pretreatment

**Figure 2:**
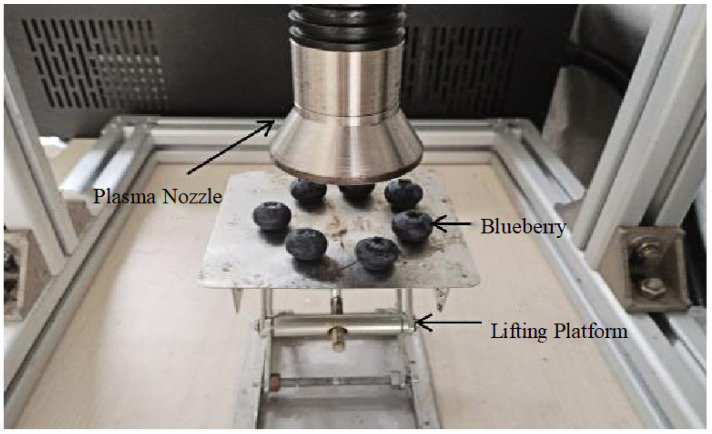
Pretreatment blueberry physical image.

During preliminary experiments, it was observed that continuous exposure of the material surface to gliding arc discharge led to a sustained temperature increase, reaching a maximum of 90 °C. Excessive temperatures caused severe surface damage to blueberries when exposed to the plasma beam for more than 20 s. Since longer pretreatment durations and higher power levels favor blueberry drying and nutrient retention, this study aimed to investigate the effects of exposure time and power on the pretreatment process. Three exposure durations were selected: 6 s, 12 s, and 18 s, with a fixed power setting of 900 W for the single-factor experiment. Given that the power of the gliding arc plasma device can be adjusted between 100 W and 900 W, three power levels were tested: 300 W, 600 W, and 900 W, with an exposure time of 18 s as the fixed parameter in this single-factor experiment. The specific pretreatment parameters are shown in Table 1, and the experimental setup is illustrated in Figure 2.

The plasma treatment was applied to the surface of blueberries using a plasma nozzle. The blueberries were positioned at a fixed distance of 10 cm from the nozzle. The treatment was localized to the outer surface of the blueberry, particularly affecting the waxy cuticle, which enhanced moisture diffusion during drying. The blueberries used in this study had an average weight of 2.00 ± 0.05 g and a length of 17.00 ± 0.30 mm. The plasma treatment was applied uniformly to the entire surface area of each blueberry, ensuring comprehensive exposure of the fruit’s cuticle to the reactive plasma species.

### 2.3. Drying Experiment Design

To ensure noticeable differences in drying rates while preventing excessively long drying times, the convective hot air drying temperature was set at a constant 65 °C [19]. Blueberries were placed on square aluminum wire mesh trays, ensuring no contact between adjacent samples. Each blueberry weighed 2.00 ± 0.05 g, with a total sample weight of 50.0 ± 0.5 g. Weight measurements were repeated three times, and the average value was recorded. Before the experiment, the drying oven was preheated for 15 min to stabilize the temperature at 65 °C. Sample weights were measured and recorded every 30 min until the moisture content reached the safe threshold of 20% [19]. The dried blueberries were then sealed in plastic wrap and stored in a −18 °C freezer for further analysis.

### 2.4. Methods for Studying Drying Kinetics

#### 2.4.1. Drying Kinetics Curve

During the drying process of the blueberries, the moisture content was determined by weighing the samples at equal time intervals [20,21]. The moisture ratio (MR) represents the variation in moisture content over drying time, and the MR–t curve was plotted accordingly [22].

The moisture ratio (MR) was calculated using Equation (1):

(1) $$MR=\frac{M_{t}-M_{e}}{M_{0}-M_{e}}$$

where *M_t_* is the moisture content at any given drying time, *M*_0_ is the initial moisture content, and *M_e_* is the equilibrium moisture content. Since *M_e_* is relatively small compared to *M_t_*, it can be neglected, simplifying Equation (2) to:

(2) $$MR=\frac{M_{t}}{M_{0}}$$

#### 2.4.2. Calculation of Moisture Diffusion Coefficient

The effective moisture diffusion coefficient (D_eff_) of blueberries during hot-air drying was determined using a simplified form of Fick’s second law of diffusion [23,24], expressed as:

(3) $$\ln MR=\ln\left(\frac{6}{\pi^{2}}\right)-\left(\frac{\pi^{2}D_{\mathrm{eff}\;}}{d^{2}}t\right)$$

where MR is the moisture ratio, D_eff_ is the effective moisture diffusion coefficient (m^2^/s), *d* is the diameter of the blueberry (m), and *t* is the drying time (s). The value of D_eff_ was determined by plotting the experimental drying data as a function of ln(MR) over time. The slope (*K*) of the resulting linear regression equation is given by:

(4) $$K=-\frac{\pi^{2}D_{\mathrm{eff}}}{d^{2}}$$

Substituting this into the equation allows for the calculation of D_eff_.

### 2.5. Anthocyanin Extraction and Analysis Methods

The method for extracting anthocyanins from dried blueberries was adapted from the procedure described by Giusti et al. [25]. Dried blueberries were first ground using a high-speed blender. One gram of blueberry pulp was then added to 25 mL of 70% methanol solution and mixed thoroughly. The extraction was performed at room temperature using ultrasound for 30 min. The extract was centrifuged at 3000 rpm for 30 min, and the supernatant was collected. This extraction process was repeated three times to obtain the final extract. Subsequently, absorbance was measured using two different buffer solutions with pH values of 4.5 and 1.0. The pH 4.5 buffer was prepared by adjusting the pH of a trisodium acetate solution, while the pH 1.0 buffer was prepared by adjusting the pH of a potassium chloride solution. For each measurement, 1 mL of anthocyanin extract was diluted to 10 mL, mixed with the corresponding buffer solution, and allowed to stand at room temperature for 15 min. Absorbance was then measured at a wavelength of 700 nm.

### 2.6. Total Phenol Extraction and Analysis Methods

The extraction method for total phenols followed the procedure described above. A 1 mL aliquot of the sample extract was transferred into a 15 mL test tube, followed by the addition of 1 mL of Folin–Ciocalteu (FC) reagent and 3 mL of 20% sodium carbonate solution. The mixture was thoroughly shaken and incubated in a water bath at 50 °C for 30 min. The absorbance was measured at 765 nm using gallic acid as the standard.

### 2.7. Color Evaluation Method

The color of dried blueberries was measured using a colorimeter. For each sample, 10 g of dried blueberries were weighed to determine their color parameters. The lightness coordinate (L*) ranges from 0 to 100, where 0 represents black and 100 represents white. The a* value represents the red–green axis, with positive values indicating red and negative values indicating green. The b* value represents the yellow–blue axis, with positive values indicating yellow and negative values indicating blue. The chroma (C) represents color saturation, ranging from gray (low values) to vivid colors (high values), where higher C values indicate more intense coloration [26]. The total color difference (ΔE) was calculated based on L*, a*, and b* values, representing the color variation between fresh and dried blueberries. A smaller ΔE indicates a closer resemblance between the dried and fresh blueberry colors [27].

The calculations were performed using the following formulas:

(5) $$C=\sqrt{a^{*2}+b^{*2}}$$

(6) $$\Delta E=\sqrt{{\left(L_{0}^{*}-L^{*}\right)}^{2}+{\left(a_{0}^{*}-a^{*}\right)}^{2}+{\left(b_{0}^{*}-b^{*}\right)}^{2}}$$

where L_0_*, a_0_*, and b_0_* represent the color parameters of fresh blueberries, while L* a*, and b* represent those of dried blueberries.

### 2.8. Rehydration Evaluation Method

The rehydration capacity of dried blueberries was evaluated following the method of Zielinska et al. [28]. Approximately 1.5 g of the dried sample was immersed in 40 mL of distilled water at room temperature for rehydration. The sample was weighed at 1 h intervals until its weight stabilized. The rehydration ratio (RR) was calculated using Equation (7):

(7) $$RR=\frac{W_{r}}{W_{d}}$$

where W_r_ and W_d_ represent the weights (g) of the rehydrated and dried samples, respectively.

### 2.9. Texture Parameter Measurement Method

The texture analysis of the dried blueberries focused on three key parameters: hardness, adhesiveness, and chewiness [29]. A TA-XT texture analyzer was used for measurements, with data processed using Exponent software. Before testing, the instrument was calibrated using a 1000 g weight. Each dried blueberry sample was placed 4 mm below the texture analyzer probe, and measurements were conducted at a test speed of 0.5 mm/s. A single dried blueberry was tested per trial, and after each test, the sample stage and probe were cleaned before the next measurement. Hardness and chewiness were analyzed based on the recorded data. For each sample group, five blueberries were tested twice, and the average value was recorded as the final result.

### 2.10. Statistical Analysis

The results were statistically analyzed using statistical software (IBM SPSS Statistics for Windows, version 25) and the data were analyzed using the one-way analysis of variance (ANOVA) and the significant differences were detected by the Tukey HSD’s test (*p* < 0.05).

## 3. Results and Discussion

### 3.1. Temperature Control and the Impact on Heat-Sensitive Compounds

In this study, we used glide arc discharge low-temperature plasma (GAD-LTP) for blueberry pretreatment. Regarding temperature control, we monitored temperature variations during the experiment, especially under conditions of extended treatment time and high power. Although the temperature was not strictly controlled during the experiment, we ensured that it did not exceed 90 °C during the pretreatment process. Preliminary experiments showed that excessively high temperatures caused severe damage to the blueberry surface, particularly when the treatment time exceeded 20 s. Therefore, in this study, we selected an 18 s pretreatment time and monitored the temperature to ensure it remained within a safe range.

During the low-temperature plasma treatment, although the temperature increased, it did not reach a level that would significantly degrade heat-sensitive compounds such as anthocyanins. According to previous studies, low-temperature plasma treatment involves minimal heat transfer, typically not inducing degradation of heat-sensitive compounds. Thus, we conclude that the impact of temperature control in this study is minimal, and the retention of heat-sensitive compounds, such as anthocyanins, is relatively good.

### 3.2. Statistical Analysis and Optimization

To further evaluate the impact of time and power on drying efficiency, we employed two-factor analysis of variance (ANOVA) and response surface methodology (RSM) for analysis and optimization.

#### 3.2.1. Analysis of Variance (ANOVA)

Initially, a two-factor analysis of variance (ANOVA) was conducted to evaluate the independent effects of power (300 W, 600 W, 900 W) and pretreatment time (6 s, 12 s, 18 s) on drying time, along with their interaction.

The ANOVA results reveal that the analysis examined the effects of power, pretreatment time, and their interaction on drying time (dependent variable). The findings indicate that both power (F = 42.95, *p* = 1.41 × 10⁻^7^) and pretreatment time (F = 126.78, *p* = 2.47 × 10⁻^11^) have significant effects on drying time, while the interaction between power and pretreatment time was not statistically significant (F = 0.83, *p* = 0.517).

Power significantly affects drying time (*p*-value = 1.41 × 10⁻^7^). Pretreatment time has an even more pronounced impact on drying time (*p*-value = 2.47 × 10⁻^11^). The interaction between power and pretreatment time does not significantly affect drying time (*p*-value = 0.517).

According to the ANOVA findings, both pretreatment time and power play significant roles in drying efficiency, with pretreatment time having the most substantial effect. Thus, optimizing both power and pretreatment time is crucial for improving drying efficiency. While both factors significantly influence drying time, their interaction is not significant. Increasing pretreatment time can significantly boost drying efficiency, with power serving as an auxiliary factor.

#### 3.2.2. Response Surface Methodology (RSM) Optimization

To further optimize the drying conditions, Response Surface Methodology (RSM) was used to model and optimize the combination of power and pretreatment time. By developing a model that describes the relationship between drying time, power, and pretreatment time, we were able to predict drying times under different conditions and identify the optimal process parameters.

**Figure 3:**
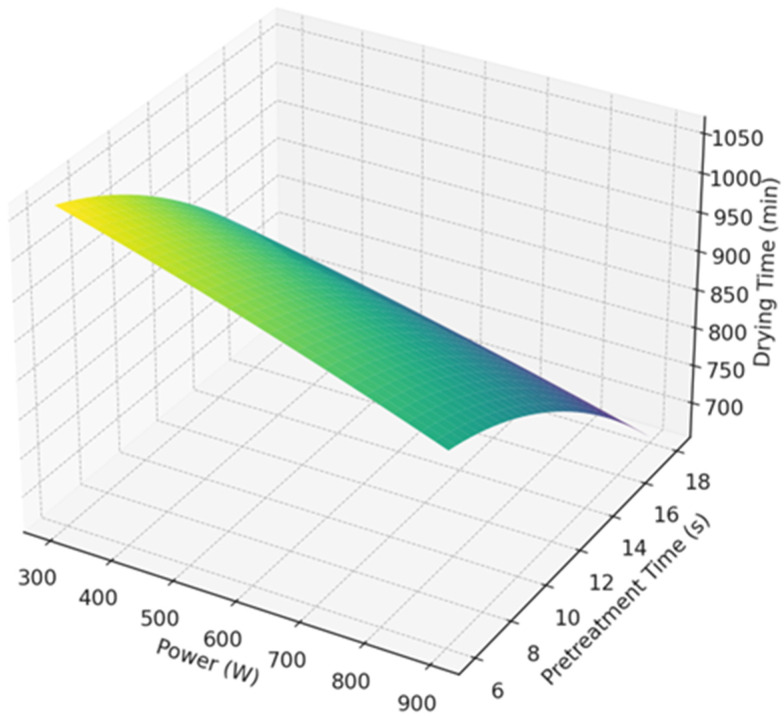
Response surface optimization—drying time vs. power and pretreatment time.

Figure 3 illustrates the relationship between drying time, power (W), and pretreatment time (s) using a response surface plot generated through response surface methodology (RSM). This 3D plot shows how the drying time (z-axis) varies as a function of both power (x-axis) and pretreatment time (y-axis).

The color gradient (from blue to red) on the surface represents the drying time, where blue indicates shorter drying times, and red indicates longer drying times. As observed in the plot, increasing pretreatment time and power generally results in a reduction of drying time, suggesting that longer pretreatment times and higher power contribute to faster drying processes. However, the relationship is not purely linear, and the interaction between these two factors influences the drying time in a non-linear manner.

The response surface highlights that pretreatment time has a more pronounced effect on the drying time compared to power. This observation aligns with the results of the two-way ANOVA, where pretreatment time was found to be the most significant factor influencing drying efficiency. This optimization model provides valuable insights into selecting the optimal power and pretreatment time settings to achieve the most efficient drying process. Specifically, higher power levels (near 900 W) combined with longer pretreatment durations (18 s) lead to the shortest drying times, suggesting that these conditions should be prioritized for maximum drying efficiency.

### 3.3. Effects on the Drying Characteristics of Blueberries

**Figure 4:**
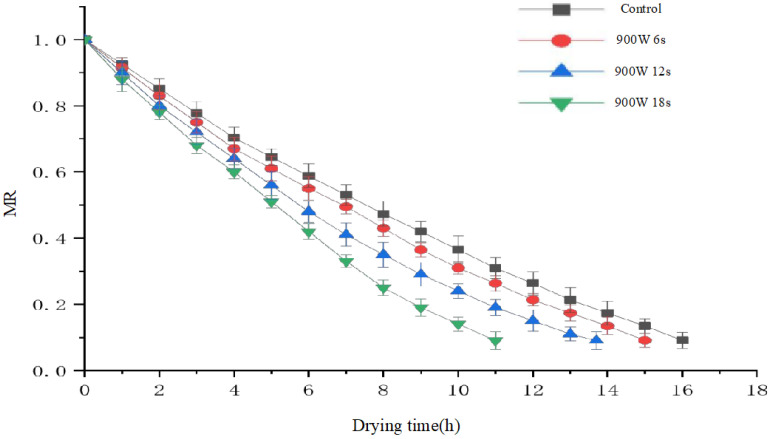
Drying curves of blueberries under different pretreatment times at 900 W power.

The effects of treatment time and power of gliding arc discharge low-temperature plasma on the drying kinetics of blueberries are illustrated in Figure 3 and Figure 4.

Figure 4 shows that when the power is fixed at 900 W (the optimal treatment power determined by preliminary experiments), increasing the drying pretreatment duration significantly reduces the total drying time. Specifically, blueberries subjected to pretreatment for 6 s, 12 s, and 18 s exhibited drying times of 15 h, 14 h, and 11 h, respectively, with a maximum reduction of 31.25%. In comparison, the control group exhibited a drying time of 16 h. This effect can be attributed to two primary factors: (1) prolonged pretreatment enhances plasma etching, facilitating moisture diffusion, which aligns with the findings of Ashtiani et al. [16]; and (2) the increased surface temperature (up to 80 °C) softens the tissue and enhances cell membrane permeability. The surface temperature was measured using a thermocouple sensor placed on the blueberry surface during treatment. The accuracy of the temperature measurements is within ±2 °C, ensuring reliable data. It should be noted that the heating effect is primarily confined to the surface of the blueberries, as the plasma energy interacts directly with the outer cuticle, which is where most of the moisture diffusion occurs. According to Pirone et al. [30], blanching improves moisture diffusion in the cherry wax layer, suggesting that gliding arc discharge low-temperature plasma may have a similar effect in increasing moisture movement from the inner layers of the blueberry to the surface.

**Figure 5:**
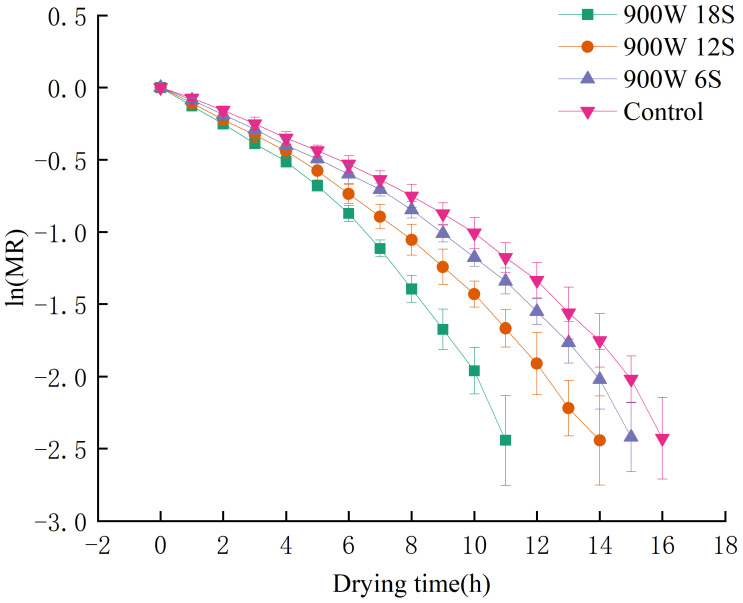
Linearized dehydration kinetics of blueberries under different pretreatment durations at 900 W power.

Figure 5 shows the linearized dehydration kinetics of blueberries under different pretreatment durations (6 s, 12 s, and 18 s) at a constant power of 900 W. The moisture ratio (MR) is plotted against the drying time (in hours), with the data for each pretreatment condition represented by distinct markers. The graph illustrates how the drying time decreases as the pretreatment duration increases, with the 18 s treatment showing the most efficient drying process. The control group (without plasma treatment) has the slowest drying kinetics, demonstrating a higher moisture ratio throughout the drying period.

**Figure 6:**
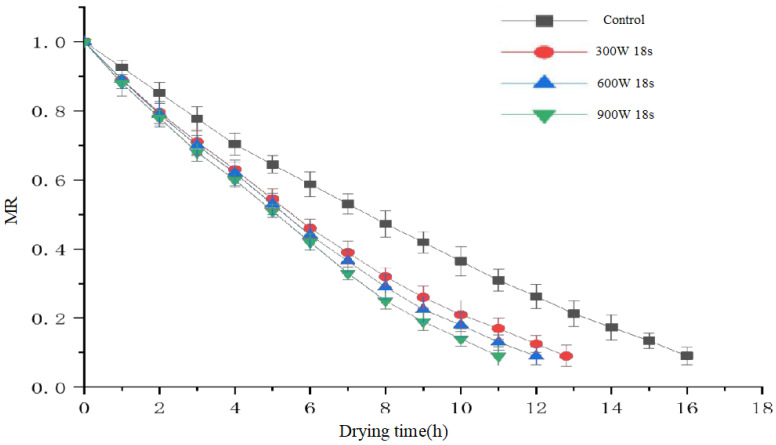
Drying curves of blueberries under different power levels with 18 s pretreatment time.

Figure 6 presents the drying kinetics of blueberries under varying treatment powers, with a fixed pretreatment time of 18 s (the optimal duration determined from preliminary experiments). The choice of 18 s as the optimal pretreatment duration was based on several factors observed during the study. First, increasing the pretreatment duration from 6 s to 18 s led to a significant reduction in drying time, as shown by the data, with the maximum reduction of 31.25% occurring at 18 s. This suggests that a longer treatment period enhances the moisture diffusion process by etching the blueberry surface more effectively, which improves drying efficiency. However, extending the treatment time beyond 18 s resulted in diminishing returns, as the additional exposure to plasma could cause excessive surface damage, adversely affecting the texture and color of the blueberries. Additionally, as the temperature during the plasma treatment can increase up to 80 °C, prolonged exposure to the plasma could also lead to the degradation of heat-sensitive compounds like anthocyanins and phenolics. Therefore, 18 s was selected as the optimal balance between improving drying efficiency and preserving the quality of the blueberries, including the retention of bioactive compounds and overall sensory attributes.

**Figure 7:**
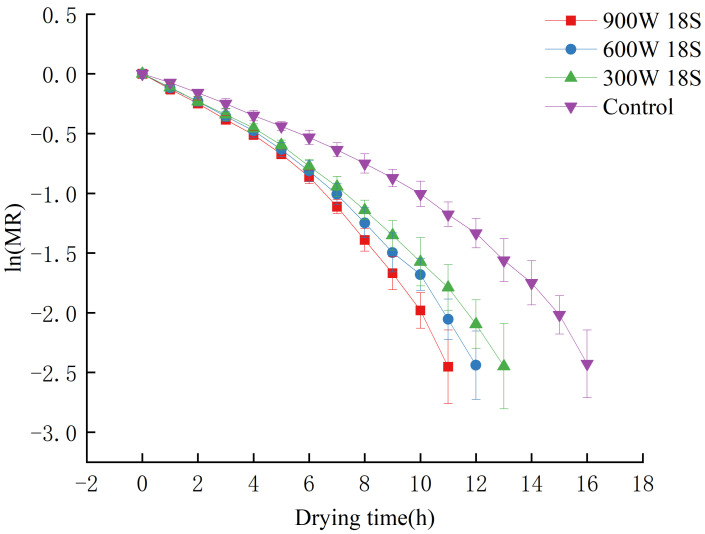
Linearized dehydration kinetics of blueberries under different power levels (300 W, 600 W, 900 W) with 18 s pretreatment.

Figure 7 presents the linearized dehydration kinetics of blueberries subjected to different pretreatment powers (300 W, 600 W, and 900 W) for 18 s. The moisture ratio (ln(MR)) is plotted against the drying time (in hours). The data show a clear trend where higher pretreatment power leads to faster drying, with the 900 W treatment exhibiting the lowest moisture ratio over time, indicating the most efficient drying process. The control group (without plasma treatment) displays the slowest drying kinetics, as indicated by the higher moisture ratio at each time point. The comparison among different power treatments highlights the impact of power on enhancing drying efficiency.

### 3.4. Effect on the Moisture Diffusion Coefficient of Dried Blueberries

The moisture diffusion coefficient during the drying process was calculated based on Fick’s second law. As shown in Table 2, the logarithm of the moisture ratio (MR) exhibited a linear relationship with drying time. When the pretreatment power was fixed at 900 W, the effective moisture diffusion coefficient of blueberries ranged from 1.2062 × 10⁻^9^ m^2^/s to 1.7040 × 10⁻^9^ m^2^/s for pretreatment durations of 6 s, 12 s, and 18 s. At a fixed pretreatment time of 18 s, increasing the power from 300 W to 900 W resulted in diffusion coefficients ranging from 1.6106 × 10⁻^9^ m^2^/s to 1.7040 × 10⁻^9^ m^2^/s. The control group had the lowest diffusion coefficient at 1.1184 × 10⁻^9^ m^2^/s. These results indicate that both longer pretreatment duration and higher power enhance moisture diffusion during blueberry drying, with pretreatment time having a more pronounced effect.

### 3.5. Effect on Anthocyanins in Dried Blueberries

Drying leads to nutrient loss in fruits and vegetables, which intensifies with prolonged drying time. While conventional pretreatment methods, such as osmotic dehydration and liquid nitrogen pretreatment, can shorten drying time, they also reduce the retention of total phenolics and anthocyanins in blueberries [31,32,33].

Table 3 presents the effects of gliding arc discharge low-temperature plasma pretreatment on anthocyanin content in dried blueberries. At a pretreatment power of 900 W, anthocyanin content initially decreased but later increased, reaching its peak at 18 s—approximately 7.9% higher than the control group. The one-way ANOVA revealed a statistically significant difference between the groups (F-statistic = 11.19, *p* = 0.00035), confirming that the increase in anthocyanin content at 18 s is statistically significant (*p* < 0.05). This trend may be attributed to anthocyanins being primarily located in the blueberry skin, where plasma exposure causes surface damage and oxidation by reactive oxygen species, leading to initial losses [34]. However, as pretreatment time increased, plasma etching accelerated drying, improving anthocyanin retention and offsetting early losses.

When pretreatment time was fixed at 18 s, different power levels (Groups 3, 4, and 5) showed a positive correlation between power and anthocyanin retention. The Tukey HSD post hoc test confirmed that the differences in anthocyanin content between the power groups were statistically significant (*p* < 0.05), with significant differences observed between Group 1 and Groups 3, 4, and 5 (*p* < 0.05), while no significant difference was found between Group 1 and Group 2 (*p* = 0.3309). This may be because higher power levels expedite drying, reducing anthocyanin degradation. Table 3 indicates that pretreatment time has a more significant impact on anthocyanin content than power level.

### 3.6. Effect on Total Phenolic Content in Dried Blueberries

The effect of gliding arc discharge low-temperature plasma treatment time and power on the total phenolic content of dried blueberries is presented in Table 3. When the pretreatment power was fixed at 900 W, total phenolic content increased with treatment time (Groups 1, 2, and 3), reaching a maximum improvement of 33.47%. One-way ANOVA revealed an F-statistic of 56.12 and a *p*-value of 6.61 × 10⁻^8^, confirming that the differences between groups were statistically significant (*p* < 0.05). This result indicates that total phenolic content increased with treatment time, particularly when the treatment time was extended to 18 s.

When the treatment time was fixed at 18 *s*, increasing power (Groups 3, 4, and 5) also led to higher total phenolic content, suggesting a positive correlation between pretreatment power and phenolic retention. The Tukey HSD post hoc test indicated that there were statistically significant differences in total phenolic content between the Control group and Groups 2, 3, 4, and 5, while no significant difference was observed between the Control group and Group 1. Specifically, the Tukey HSD post hoc test showed significant differences between the Control group and Group 3, Control group and Group 4, and Control group and Group 5 (*p* < 0.05), while there was no significant difference between the Control group and Group 1 (*p* = 0.9668).

This effect may be attributed to the reduction in drying time, which minimizes prolonged exposure to high temperatures and mitigates phenolic degradation. Table 3 indicates that treatment time had a greater impact on total phenolic content than treatment power.

### 3.7. The Effect on the Color of Dried Blueberries

**Figure 8:**
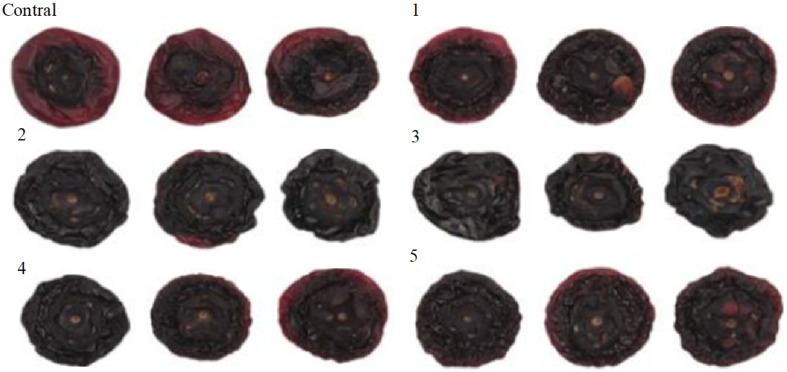
Appearance of blueberries after drying in different treatment groups. Appearance of the control group and experimental groups 1–5.

The appearance changes of dried blueberries after different pretreatment parameters are shown in Figure 8. The effects of different pretreatment parameters on the color of dried blueberries are shown in Table 4. Lightness (L*) reflects the degree of browning during the drying process, with the control group exhibiting the highest L* value. Both pretreatment power and duration negatively correlated with L*, with duration having a more significant impact. Chroma (C) represents color intensity, with untreated dried blueberries displaying the highest C value. Pretreatment power and duration negatively correlated with C, with duration being the dominant factor. Color difference (ΔE) indicates overall color variation, with the control group showing the lowest ΔE value. Both power and duration positively correlated with ΔE, with duration having a stronger effect, leading to more pronounced color changes.

Lightness (L*) reflects the degree of browning during the drying process, with the control group exhibiting the highest L* value. One-way ANOVA revealed an F-statistic of 21.40 and a *p*-value of 1.35 × 10⁻^5^, confirming that the differences in L* values between the groups were statistically significant (*p* < 0.05). Both pretreatment power and duration negatively correlated with L*, with duration having a more significant impact. Tukey HSD post hoc test further showed significant differences between the Control and Groups 3, 4, and 5 (*p* < 0.05), indicating that longer treatment times and higher power levels contributed to a greater decrease in L*. Specifically, the Control group was significantly different from Group 3, Group 4, and Group 5, while there was no significant difference between the Control group and Group 1 or Group 2.

Chroma (C) represents color intensity, with untreated dried blueberries displaying the highest C value. One-way ANOVA showed an F-statistic of 3.42 and a *p*-value of 0.0376, confirming that pretreatment time and power significantly affected the C values (*p* < 0.05). Both pretreatment power and duration negatively correlated with C, with duration being the dominant factor. Tukey HSD post hoc test revealed significant differences between the Control and Groups 2, 3, 4, and 5 (*p* < 0.05), indicating that longer pretreatment times and higher power levels reduced the color intensity of the blueberries.

Color difference (ΔE) indicates overall color variation, with the control group showing the lowest ΔE value. One-way ANOVA indicated an F-statistic of 32.70 and a *p*-value of 1.37 × 10⁻⁶, confirming significant differences in ΔE values between groups (*p* < 0.05). Both power and duration positively correlated with ΔE, with duration having a stronger effect, leading to more pronounced color changes. Tukey HSD post hoc test showed significant differences between the Control group and Groups 4 and 5 (*p* < 0.05), indicating that longer pretreatment times and higher power levels caused more noticeable color changes. Specifically, Group 4 and Group 5 exhibited significantly higher ΔE values than the Control group, while Group 1 and Group 2 did not show significant differences.

Unlike Ashtiani et al.’s study, which found that low-temperature plasma treatment enhanced C and reduced ΔE in grapes [16], this study observed the opposite trend in blueberries. Possible explanations for this discrepancy include: (1) prolonged treatment and higher power intensifying surface etching, leading to epidermal abrasion and reduced brightness; (2) ozone and other strong oxidizing agents produced by plasma exacerbating browning [35]. These factors contribute to a reduction in the visual appeal of the blueberries, potentially altering their attractiveness to consumers.

However, it is crucial to consider the trade-off between drying efficiency and color quality. The primary advantage of low-temperature plasma treatment is its ability to accelerate the drying process, leading to significantly higher drying efficiency. This is particularly beneficial in large-scale industrial applications, where reducing drying time directly translates to lower energy consumption and processing costs. By shortening the drying time, the process becomes more cost-effective, helping to meet the demands of large-scale production while minimizing resource usage. Moreover, the reduction in processing time helps preserve certain volatile compounds that could otherwise degrade during prolonged drying, further enhancing the efficiency of the process.

On the other hand, the observed decrease in brightness (L*) and color intensity (C), along with the increase in color difference (ΔE), may affect consumer perception to some extent. However, the potential benefits of low-temperature plasma treatment—such as higher drying efficiency and better nutrient retention—significantly outweigh the minor negative impact on color quality. While the appearance of fruits is an important quality attribute, the accelerated drying process provided by plasma treatment offers considerable advantages for both cost-effectiveness and nutritional value preservation. These advantages are especially crucial in large-scale food processing where time, energy, and cost efficiency are key factors.

Furthermore, while more pronounced color changes, especially increased browning, could lower the visual appeal of the blueberries to some extent, the nutrient retention (such as anthocyanins and total phenolics) remains superior compared to traditional methods. This makes the blueberries more valuable from a health perspective, providing consumers with a higher antioxidant content, which is a growing concern in the market. For instance, the accelerated drying process minimizes nutrient degradation, especially in the context of antioxidants that are sensitive to prolonged heat exposure.

Therefore, while low-temperature plasma treatment does result in some color changes—primarily in terms of a decrease in brightness and color intensity—the overall benefits, particularly improved drying efficiency and nutrient retention, make it a more favorable option. By carefully optimizing treatment time and power, it is possible to achieve an ideal plasma treatment that maximizes drying efficiency while minimizing undesirable color changes. This balance is critical not only for improving the competitiveness of plasma-treated blueberries in the market but also for ensuring the product meets both technological requirements and consumer health expectations. As such, the benefits of the plasma treatment far outweigh its minor negative impact on color quality, and the process can be further optimized to enhance both efficiency and product appeal.

### 3.8. Effect of Pretreatment on the Rehydration Capacity of Dried Blueberries

**Figure 9:**
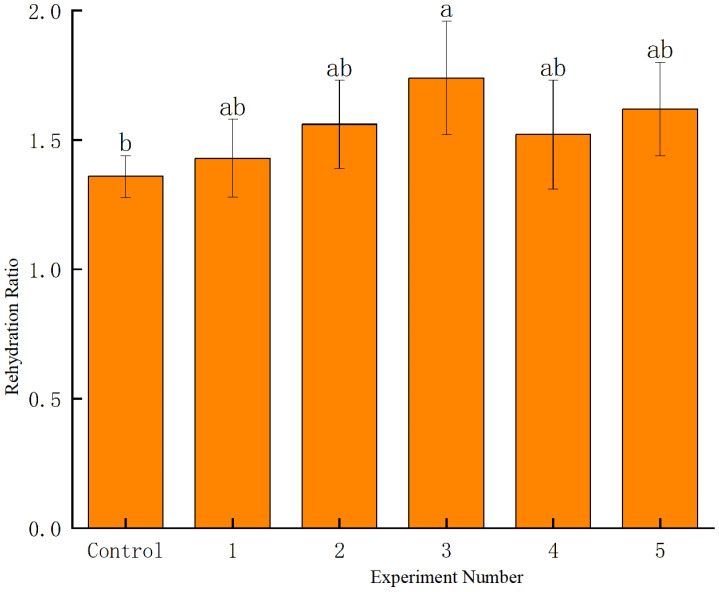
Dried blueberry rehydration under different time gradients. Data are mean ± standard deviation (SD) of three replicates. Different letters are significantly different using ANOVA at *p* < 0.05.

Rehydration capacity reflects the impact of drying on the material’s microstructure, with lower rehydration indicating greater structural damage [36]. Figure 9 illustrates the effects of different gliding arc discharge cold plasma treatment conditions on the rehydration capacity of dried blueberries. At a constant pretreatment power, rehydration increased with treatment duration, reaching a 27.94% improvement at 18 s. Similarly, at a fixed treatment duration, higher power enhanced rehydration, with a maximum increase of 19.12%. This effect may be attributed to (1) plasma etching creating micropores, facilitating water diffusion, and (2) reduced drying time, minimizing structural damage to the blueberry surface.

### 3.9. Effects on the Texture of Dried Blueberries

Hardness reflects the firmness of dried blueberries, with higher values indicating greater hardness. Adhesiveness represents the intercellular bonding force, where higher values suggest a denser tissue structure. Chewiness quantifies the force required for chewing, with larger values indicating greater chewiness [37]. Table 5 presents the effects of gliding arc discharge low-temperature plasma treatment on the texture of dried blueberries.

At a constant pretreatment power, hardness increased with treatment duration, showing a maximum increase of 35.49%. In contrast, adhesiveness and chewiness decreased, with maximum reductions of 25.89% and 27.23%, respectively. One-way ANOVA for hardness revealed an F-statistic of 4.01 and a *p*-value of 0.0226, confirming that the differences in hardness between the groups were statistically significant (*p* < 0.05). For adhesiveness, the F-statistic was 21.94 with a *p*-value of 1.18 × 10⁻^5^, indicating highly significant differences between the groups (*p* < 0.05). Similarly, the ANOVA for chewiness resulted in an F-statistic of 5.95 and a *p*-value of 0.0054, confirming significant differences between the groups (*p* < 0.05).

The possible reasons for these changes include: (1) Reactive species in the plasma (such as free radicals, atoms, and ions) reacting with polysaccharides and proteins in the cell wall, altering their chemical composition and physical properties, thereby affecting texture [38]. (2) Plasma treatment influences enzymatic activity in fruits and vegetables, leading to changes in the activity of enzymes like pectinase, cellulase, and polyphenol oxidase, which, in turn, affect the texture of dried blueberries [39]. (3) Plasma etching creates micropores, enhancing moisture diffusion and accelerating drying. This results in volume shrinkage and a denser texture, while rapid moisture evaporation can cause surface contraction, leading to a firmer outer skin [40].

Tukey HSD post hoc test further showed significant differences between the Control group and Groups 3, 4, and 5 (*p* < 0.05), indicating that longer treatment times and higher power levels contributed to greater increases in hardness and decreases in adhesiveness and chewiness. Specifically, the Control group was significantly different from Group 3, Group 4, and Group 5, while there was no significant difference between the Control group and Group 1 or Group 2.

At a constant treatment duration, hardness increased with higher pretreatment power, whereas adhesiveness decreased. The control group exhibited the highest adhesiveness and chewiness.

### 3.10. Energy Efficiency of GAD-LTP and Comparison with Other Pretreatment Methods

This study focuses on the impact of low-temperature plasma pretreatment (GAD-LTP) on the drying efficiency and quality of blueberries. While the primary focus is on optimizing the final product quality, energy efficiency is also a crucial factor that offers a more comprehensive perspective on the industrial application of this technology. The following provides an analysis of the energy efficiency of GAD-LTP and a comparison with other pretreatment methods:

GAD-LTP is a low-temperature, efficient pretreatment method that uses reactive oxygen species (such as O_3_ and OH) generated by arc discharge to treat the blueberry surface, facilitating moisture diffusion. Throughout the process, the energy consumption of low-temperature plasma remains relatively low, and the method does not require high-temperature heating, ensuring a balance between processing efficiency and energy efficiency. Compared to traditional thermal treatments, GAD-LTP not only completes the treatment in a shorter time but also avoids nutrient loss due to excessive heat exposure. Specifically, low-temperature plasma treatment typically maintains a temperature range of 30 °C to 90 °C, which is much lower than the high temperatures (e.g., above 150 °C) required for traditional thermal treatments. As a result, the energy consumption of GAD-LTP is significantly lower than that of high-temperature drying methods. Additionally, the short treatment time further enhances the overall energy efficiency of the process.

Conventional thermal methods, particularly hot air drying, often require extended periods at high temperatures, which consume a large amount of energy and can degrade heat-sensitive compounds in blueberries, such as anthocyanins and total phenols. Hot air drying generally results in high energy consumption, and prolonged heat exposure can reduce drying efficiency, increasing production costs. In contrast, GAD-LTP utilizes low-temperature plasma technology to significantly reduce drying time without requiring high-temperature heating, while maintaining high drying efficiency and preserving nutrient content. This provides GAD-LTP with a distinct advantage in terms of energy efficiency and quality retention.

Ultrasound pretreatment, which accelerates moisture diffusion and mass transfer through high-frequency sound waves, can improve drying rates. However, the equipment cost for ultrasound treatment is relatively high, and the method’s energy consumption is significant, particularly in large-scale industrial applications where energy efficiency becomes a key limiting factor. GAD-LTP, on the other hand, demonstrates superior energy efficiency. While ultrasound pretreatment can accelerate the drying process, its high energy consumption results in lower overall energy efficiency. By contrast, GAD-LTP operates efficiently by using low-temperature plasma to treat the blueberry surface in a short time, offering better energy efficiency and lower operational costs, making it more suitable for industrial-scale applications.

Freeze drying is an effective method for preserving food nutrients and flavor but is both time-consuming and energy-intensive. The freezing phase, in particular, consumes significant energy, and both equipment investment and maintenance costs are high. In comparison, GAD-LTP provides significant advantages in energy efficiency. It does not require extremely low temperatures and can complete pretreatment in a short time, thus avoiding the energy wastage associated with freeze drying. This makes it a more economically viable option for large-scale industrial production.

Although this study has not conducted a comprehensive quantification of GAD-LTP’s energy efficiency, preliminary results suggest that GAD-LTP is associated with low energy consumption and high drying efficiency. Future research will focus on evaluating GAD-LTP’s energy efficiency in greater detail, including comparisons with other pretreatment methods (such as hot air drying, ultrasound treatment, and freeze drying), quantifying energy consumption under various conditions, and optimizing pretreatment parameters to maximize energy efficiency.

Comparing GAD-LTP with other pretreatment methods reveals its high energy efficiency, ability to improve drying efficiency, and capacity to preserve food quality. These attributes make GAD-LTP particularly well-suited for industrial applications. While current research has not fully assessed its energy efficiency, future studies will provide a more detailed evaluation, offering additional theoretical support and practical insights for its application.

## 4. Conclusions and Discussion

Gliding arc discharge low-temperature plasma (GAD-LTP) pretreatment significantly impacts the drying kinetics and quality of dried blueberries. It accelerates the drying process by enhancing moisture diffusion, which reduces processing time. This leads to improved retention of total phenolics and anthocyanins, both of which contribute to the nutritional quality of blueberries. The enhanced rehydration capacity suggests that, despite accelerated drying, the structural integrity of the blueberries is better preserved than with traditional methods. Both pretreatment time and power are positively correlated with the drying rate and rehydration capacity, with time exerting a more significant effect. Increasing these parameters improves the retention of total phenolics and anthocyanins. However, it should be noted that while these changes are beneficial nutritionally, longer treatment times and higher power levels negatively impact color and texture. Specifically, both pretreatment time and power negatively correlate with lightness (L*), chroma (C), and total color difference (ΔE), with extended treatment causing more pronounced deterioration in color quality.

The optimal condition of 18 s and 900 W was chosen because it achieved the highest retention of anthocyanins and total phenolics while also yielding superior drying characteristics. These findings demonstrate that GAD-LTP is a promising, scalable pretreatment method for blueberry drying, offering a favorable balance between process efficiency and quality preservation. However, it is essential to recognize the trade-offs between drying efficiency, nutrient retention, and sensory quality—especially regarding color and texture.

Although the 18 s and 900 W treatment condition resulted in the highest retention of key nutrients, including anthocyanins and total phenolics, the negative impact on color and texture should not be overlooked. This highlights the need for careful optimization of treatment parameters. In this study, drying efficiency and nutrient preservation—critical for large-scale production—were prioritized over sensory quality, which can be adjusted with further refinement of the treatment process. The decision to consider 18 **s** and 900 W as optimal is based on the substantial benefits in drying efficiency and nutrient retention, which outweigh the moderate color degradation. Nutritional preservation and energy-efficient processing are essential in industrial applications, where maintaining high throughput and reducing costs are key objectives.

In practice, the increased drying efficiency achieved through plasma treatment reduces overall processing time, minimizing energy consumption and improving the cost-effectiveness of the drying process. While this often leads to some color changes, such as increased browning, the trade-off can be justified, particularly in situations where nutrient preservation and reduced drying time are of greater importance to manufacturers. Furthermore, the slight color degradation observed under optimal conditions is unlikely to deter consumers who prioritize nutritional value and overall product quality, especially in markets increasingly focused on health-conscious choices.

However, while the improvements in drying efficiency and nutrient retention are promising, future research should also address the long-term storage stability, microbial safety, and shelf-life of the dried blueberries. These factors are critical for industrial scalability and the commercial viability of GAD-LTP-treated products. Ensuring that the dried blueberries maintain their quality over extended storage periods, remain microbiologically safe, and meet regulatory standards for shelf-life will be essential for widespread adoption in the food industry.

Future studies should focus on minimizing color degradation while continuing to maintain high drying efficiency and nutrient retention at 18 s and 900 W. Further optimization of treatment parameters, such as adjusting treatment time and power for different fruit types or exploring lower power settings, could help balance the trade-off between color quality and efficiency. Expanding this technology to other fruits and optimizing plasma parameters for a variety of crops will enhance its applicability across the food industry. By addressing these sensory quality concerns and incorporating long-term stability and safety considerations, future studies could refine treatment conditions to strike a better balance between quality preservation, consumer acceptance, processing efficiency, and product durability.

## Figures and Tables

**Table 1:** Experimental grouping.

Group Number	Treatment Time (s)	Power (W)
1	6	900
2	12	900
3	18	900
4	18	300
5	18	600

**Table 2:** Table of effective diffusion coefficient of dried water of blueberry under different pretreatment parameters.

Group Number	First-Order Regression Equation	R^2^	D_eff_/(10^−9^ m^2^s^−1^)
Control group	lnMR = −3.82 × 10^−5^ t + 0.2004	0.9516	1.1184
1	lnMR = −4.12 × 10^−5^ t + 0.1855	0.9598	1.2062
2	lnMR = −4.76 × 10^−5^ t + 0.1842	0.9732	1.3949
3	lnMR = −5.81 × 10^−5^ t + 0.2068	0.9589	1.7040
4	lnMR = −5.50 × 10^−5^ t + 0.1801	0.9748	1.6106
5	lnMR = −5.72 × 10^−5^ t + 0.1731	0.9787	1.6752

**Table 3:** Physicochemical properties of dried blueberry under different pretreatment parameters. Data are mean ± standard deviation (SD) of three replicates. Different letters are significantly different using ANOVA at *p* < 0.05.

Group Number	Anthocyanin Content (mg/100 g)	Total Phenolic Content (mg/g)
Control group	8.20 ± 0.12 ^a^	160.07 ± 2.31 ^a^
1	7.81 ± 0.22 ^ab^	167.45 ± 3.66 ^a^
2	8.16 ± 0.21 ^b^	172.43 ± 5.12 ^b^
3	8.85 ± 0.18 ^c^	213.65 ± 6.32 ^c^
4	8.56 ± 0.24 ^bc^	199.83 ± 4.42 ^d^
5	8.68 ± 0.21 ^c^	205.32 ± 5.21 ^e^

**Table 4:** Effect of gliding arc discharge plasma pretreatment on dry color of blueberry. Data are mean ± standard deviation (SD) of three replicates. Different letters are significantly different using ANOVA at *p* < 0.05.

Group Number	L*	*C*	Δ*E*
Control group	22.23 ± 0.48 ^a^	0.89 ± 0.05 ^a^	2.67 ± 0.22 ^a^
1	21.79 ± 0.43 ^a^	0.83 ± 0.12 ^d^	2.83 ± 0.25 ^a^
2	20.52 ± 0.51 ^b^	0.72 ± 0.11 ^d^	3.07 ± 0.23 ^a^
3	18.11 ± 0.73 ^c^	0.58 ± 0.14 ^b^	4.66 ± 0.23 ^b^
4	19.12 ± 0.75 ^c^	0.63 ± 0.18 ^c^	4.97 ± 0.52 ^c^
5	18.66 ± 0.82 ^c^	0.61 ± 0.21 ^c^	4.81 ± 0.45 ^c^

**Table 5:** Effect of pretreatment in a gliding arc discharge plasma treatment unit on the dry texture of blueberries. Data are mean ± standard deviation (SD) of three replicates. Different letters are significantly different using ANOVA at *p* < 0.05.

Group Number	Hardness (g)	Adhesiveness (g)	Chewiness (mJ)
Control group	220.32 ± 13.64 ^a^	85.32 ± 5.23 ^a^	2.46 ± 0.15 ^a^
1	235.63 ± 18.21 ^a^	83.23 ± 4.23 ^a^	2.22 ± 0.19 ^a^
2	260.33 ± 27.65 ^a^	70.52 ± 2.62 ^b^	1.83 ± 0.21 ^b^
3	298.52 ± 33.78 ^b^	63.23 ± 1.42 ^b^	1.79 ± 0.23 ^b^
4	277.73 ± 31.45 ^b^	71.64 ± 2.14 ^b^	1.61 ± 0.32 ^b^
5	290.21 ± 29.76 ^b^	80.23 ± 1.33 ^a^	1.72 ± 0.27 ^b^

## Data Availability

The original contributions presented in this study are included in the article. Further inquiries can be directed to the corresponding authors.
